# Reply to Naderi Beni et al. Comment on “Ulas et al. Prognostic Insights into Orbital Metastases: A Comprehensive Analysis of Clinical Features and Survival Outcomes. *Diagnostics* 2025, *15*, 2542”

**DOI:** 10.3390/diagnostics16101536

**Published:** 2026-05-19

**Authors:** Burak Ulas, Altan Atakan Ozcan, Feyza Alara Celikten, Omer Kaya, Ertugrul Bayram

**Affiliations:** 1 Department of Ophthalmology, Faculty of Medicine, Çukurova University, Adana 01330, Türkiye; 2 Department of Radiology, Faculty of Medicine, Çukurova University, Adana 01330, Türkiye; 3 Department of Medical Oncology, Faculty of Medicine, Çukurova University, Adana 01330, Türkiye

We would like to thank the authors of the comment [1] for their interest in our study and for their thoughtful and constructive observations [2].

We agree that, as we mentioned in the limitations section, given the relatively small overall cohort and particularly limited subgroup sizes for certain primary tumors, performing multivariable survival analyses or detailed histopathological/molecular subtyping would have been statistically inappropriate and potentially prone to overfitting. For this reason, we deliberately adopted a cautious and descriptive analytical approach.

Regarding the absence of carcinoma of unknown primary in our series, we believe this finding reflects the tertiary referral nature of our center and the routine use of extensive systemic diagnostic work-up, rather than selection bias, and is consistent with reports from specialized orbital centers.

Finally, as acknowledged in our manuscript, heterogeneity of treatment modalities and limited statistical power preclude definitive prognostic conclusions, and our results should be interpreted accordingly.

We appreciate the opportunity to further clarify these points.
